# Draft Genome Sequence of *Propionibacterium avidum* Strain UCD-PD2 Isolated from a Feline Anal Sac

**DOI:** 10.1128/genomeA.00034-17

**Published:** 2017-03-23

**Authors:** Petra A. Dahms, Alexandra L. Martin, Holly H. Ganz, Jonathan A. Eisen, David A. Coil

**Affiliations:** a University of California Davis Genome Center, Davis, California, USA; b Department of English Literature, University of California Berkeley, Berkeley, California, USA; c Department of Evolution and Ecology and Department of Medical Microbiology and Immunology, University of California Davis, Davis, California, USA

## Abstract

Here, we present the draft genome sequence of *Propionibacterium* (*Cutibacterium*) *avidum* strain UCD-PD2. The assembly contains 2,667,287 bp in 51 contigs. The strain was isolated from anal sac secretion samples collected from a feral domestic cat (*Felis catus*) as part of a larger project to study the microbiology of cats.

## GENOME ANNOUNCEMENT

Cats use scent marking to mark their territory and attract mates. Anal sacs found on the sides of the rectum release a pungent liquid into a cat's stool, allowing other cats to recognize its territory (1). We hypothesize that the distinct odors associated with anal sac secretions are produced by bacteria living within the anal sacs. The goal of this project was to isolate and characterize anal sac bacterial isolates from the domestic cat, *Felis catus*.

*Propionibacterium* (*Cutibacterium*) *avidum* is an anaerobic Gram-positive bacterium primarily found on moist areas of human skin (2), recently proposed for reclassification as *Cutibacterium avidum* (3). *P. avidum* UCD-PD2 was isolated from feline anal sac secretions as part of a larger study on cat microbiology (kittybiome). Anal sacs from sedated feral cats were expressed as part of a spay and neuter clinic. Swab samples of these secretions were added to 1× phosphate-buffered saline (PBS). Fifty microliters of this mixture was inoculated onto Columbian blood agar plates and incubated under anaerobic conditions (BD GasPak EZ container system) at 37°C for 7 days. A single colony was selected and subcultured on Columbian blood agar plates for 5 days at 37°C. This process of selection, isolation, and growth under these conditions was repeated three times. A Promega Wizard genomic DNA purification kit was used for DNA extraction from colonies scraped from blood agar plates. Colony PCR was performed to amplify the 16S rRNA gene using 27F and 1391R primers, sequenced using Sanger sequencing, and analyzed using BLAST (4). An alignment between *P. avidum* UCD-PD2 and other *Propionibacterium* spp. was created using the Ribosomal Database Project (RDP) (5). An approximate maximum likelihood phylogenetic tree was created using FastTree and viewed in Dendroscope (6, 7). *P. avidum* UCD-PD2 was found in a clade containing other *Propionibacterium* strains.

A paired-end library was made using a Nextera XT library preparation kit (Illumina) in preparation for whole-genome sequencing. Fragments of 600 to 900 bp were selected using a Pippin Prep (SageScience). The size-selected library was sequenced on a paired-end 300-bp run of Illumina MiSeq.

After quality trimming and error correction by the A5-miseq assembly pipeline, 642,282 high-quality reads were processed into 51 resulting contigs (longest contig, 440,561 bp; *N*_50_, 115,268 bp) (8, 9). These contigs were submitted to GenBank. The final assembly of UCD-PD2 had 2,662,308 bp, with a G+C content of 63.3% and overall coverage of 31×. Genome completeness was estimated using the PhyloSift software, which searched for 37 highly conserved single-copy marker genes, and one copy of each was found in this assembly (10).

Annotation was performed using RAST (11). *P. avidum* UCD-PD2 contains 2,478 coding sequences and 51 noncoding RNAs. The full-length 16S rRNA sequence (1,518 bp) was analyzed as described above. *P. avidum* UCD-PD2 was found in a clade containing mostly *P. avidum* sequences and a sole *Propionibacterium propionicum* sequence as the phylogenetically closest relative. However, further analysis showed that this *P. propionicum* sequence is misidentified and belongs to *P. avidum*. This misidentification was confirmed with both RDP and DSMZ, from whence the *P. propionicum* culture came (B. Chai [RDP], R. Pukall [DSMZ], personal communications).

### Accession number(s).

This whole-genome shotgun project has been deposited at DDBJ/ENA/GenBank under the accession no. LYSN00000000. The version described in this paper is version LYSN01000000.
